# Risk Levels of Invasive *Fusarium oxysporum* f. sp. in Areas Suitable for Date Palm (*Phoenix dactylifera*) Cultivation under Various Climate Change Projections

**DOI:** 10.1371/journal.pone.0083404

**Published:** 2013-12-10

**Authors:** Farzin Shabani, Lalit Kumar

**Affiliations:** Ecosystem Management, School of Environmental and Rural Science, University of New England, Armidale, New South Wales, Australia

## Abstract

Global climate model outputs involve uncertainties in prediction, which could be reduced by identifying agreements between the output results of different models, covering all assumptions included in each. *Fusarium oxysporum* f.sp. is an invasive pathogen that poses risk to date palm cultivation, among other crops. Therefore, in this study, the future distribution of invasive *Fusarium oxysporum* f.sp., confirmed by CSIRO-Mk3.0 (CS) and MIROC-H (MR) GCMs, was modeled and combined with the future distribution of date palm predicted by the same GCMs, to identify areas suitable for date palm cultivation with different risk levels of invasive *Fusarium oxysporum* f.sp., for 2030, 2050, 2070 and 2100. Results showed that 40%, 37%, 33% and 28% areas projected to become highly conducive to date palm are under high risk of its lethal fungus, compared with 37%, 39%, 43% and 42% under low risk, for the chosen years respectively. Our study also indicates that areas with marginal risk will be limited to 231, 212, 186 and 172 million hectares by 2030, 2050, 2070 and 2100. The study further demonstrates that CLIMEX outputs refined by a combination of different GCMs results of different species that have symbiosis or parasite relationship, ensure that the predictions become robust, rather than producing hypothetical findings, limited purely to publication.

## Introduction

Climatic parameters including temperature, moisture, windiness and snowfall play an important role in the development of associations between crops and diseases [1]. However, studies on the implication of climate change on future distribution of plant diseases continue to be restricted. Coakley et al. [2] concluded that climate alteration could change the geographical distribution of host and pathogen, and changes could be different for different pathosystems. Further, Chakraborty et al. [3] documented that alterations in temperature and precipitation may cause relocation of crops and plant diseases. Moreover, Singh et al. [4] reported that climate alteration will change rates of oil palm diseases. In addition, Paterson [5] demonstrated that climate change will impose negative effects on oil palm trees because of greater stresses caused by climate alteration. Paterson [5] also concluded that the distribution of oil palm diseases will change as climate changes. Rosenzweig et al. [6] modeled results on the impact of climate change on the yield of four different crops, wheat, maize, rice and soybean, and showed a significant reduction with a 4 °C increase in global temperature. These cash crops may continue to be cultivated in marginal climates for economic purposes, and diseases, which are now inactive due to unfavorable climatic condition, may rapidly become serious problems under altering climate. Podger et al. [7] documented that the negative effects of *Phytophthora cinnamomi* on *Eucalyptus spp.* on forests in Tasmania were negligible due to low ambient temperatures; however, increase in soil temperatures and wet periods could provide favorable conditions for this disease in southeast Australia [3]. Moreover, the literature shows that a change to warmer climate makes favorable condition for *Melampsora alli-populina* whose pathogen is a serious threat to poplar clones [1]. Therefore, studying the effect of climate change on future distribution of economic crops and their diseases, is as much about detecting new opportunities as about minimizing undesirable effects. 

*Fusarium oxysporum* f.sp. is an invasive pathogen responsible for wilt and cortical rot diseases in more than a hundred cash crops, including date palm (*Phoenix dactylifera* L.) [8,9]. Bayoud disease, caused by this pathogen, destroyed two-thirds of date palm in North Africa, especially in Morocco (more than 10 million trees) and in the western and central parts of Algeria in 20^th^ centuries [10,11]. *Fusarium oxysporum* f.sp. was also reported as a potential danger to date production in California [12]. It is reported that *Fusarium* spp. was the main cause of disease in *P.canariensis* and other palm species in the Canary Islands [13]. The most distinguishing sign of this lethal disease is an asymmetrical wilt of the leaflets on one side of the rachis. The wilt progresses from the rachis base to the rachis apex and, after this stage, the other side of the rachis begins to wilt [13]. *Fusarium oxysporum* f.sp. has also been recognized as the main cause of substantial crop losses in Egypt, Sudan, Israel, Brazil and Paraguay [14]. It is documented that in India, a yield loss of 80-100% of tuberous crops is associated with the *Fusarium oxysporum* species [15,16]. Therefore, modeling the future distribution of *Fusarium oxysporum* f.sp. may be useful in making advanced informed choices regarding suitable locations for the economic production of date palm and associated industries.

Sustainable cash crop production requires long-term management strategies, which are dependent on information derived from current and future climate scenarios, such as anticipated distribution and relative abundance. This issue has been addressed by bioclimatic models, ecological niche models (ENMs) and species distribution models (SDMs) [17-19]. Another model frequently used in prediction and assessment of climate change impact on different species is BIOCLIM [20]. However a major criticism of BIOCLIM is that the use of all 35 variables leads to an ‘over-fitting’ of the model, which may create misrepresentations of future species’ distribution [20]. Other fields in which global climate models (GCMs) are regarded as useful tools are invasive species management, conservation, ecology, biogeography and evolution [21]. Biogeography, conservation biology and environmental management studies have, since 1996, used GCMs extensively [19,22-28]. The main purpose of climatic models is to determine the sensitivity of plants, diseases and invasive species to climate alteration [22]. Species’ tolerance limits are derived using the current climate range to establish climate conditions and thereafter changes in suitable areas for species growth are projected into the future, using different climate change scenarios [29]. There is a growing body of literature confirming that CLIMEX is the most suitable software to model species distribution into the next century [28,30-37].

The CLIMEX model is defined as semi-mechanistic and allows users to predict future geographical distribution of different species [32,38]. The eco-physiological growth model forms the basis of the CLIMEX model, with a basic premise that favorable seasons lead to positive growth, while unfavorable seasons produce negative growth in any specific population [23,32,39-41]. Thus CLIMEX facilitates the employment of climate mapping to create the potential niche of particular species and pests within an alien range [33]. A major criticism of CLIMEX is that it does not include biotic interactions and dispersal in the modeling process [42,43]. However, it has been recommended that interpolating additional non-climatic factors such as land use, soil type, biotic interaction, diseases, and competition makes CLIMEX results more accurate and robust, since the results have to satisfy other requirements [44-46].

Quantification of the impact of climate change is a prerequisite for deliberation on both long-term and short-term countermeasures to reduce the impact of climate change. Shabani et al. [28] have recently produced data on the global distribution of date palm and the areas suitable for its cultivation. This paper is about modeling the risk of date palm cultivation from *Fusarium oxysporum* f.sp. for the same years as the date palm projections of Shabani et al. (2012). Therefore, we modeled the future distribution of *Fusarium oxysporum* f.sp. and overlayed these onto the future predicted distribution of date palm to determine which areas are would be highly conducive to date palm cultivation but at the highest risk, medium and the lowest risk of *Fusarium oxysporum* f.sp.. In this study, attempts were made to improve the projections to be directed to enhance accuracy by finding the common projected areas by both GCMs for both date palm and *Fusarium oxysporum* f.sp.. Here, CLIMEX software was utilized to simulate a model of the response of *Fusarium oxysporum* f.sp. to climate change. ArcGIS software was used to extract the CLIMEX outputs for projecting the suitable areas for date palm cultivation under different risk levels of invasive *Fusarium oxysporum* f.sp. in future. 

## Materials and Methods

### Current distribution of *Fusarium oxysporum* f.sp

**Figure 1:**
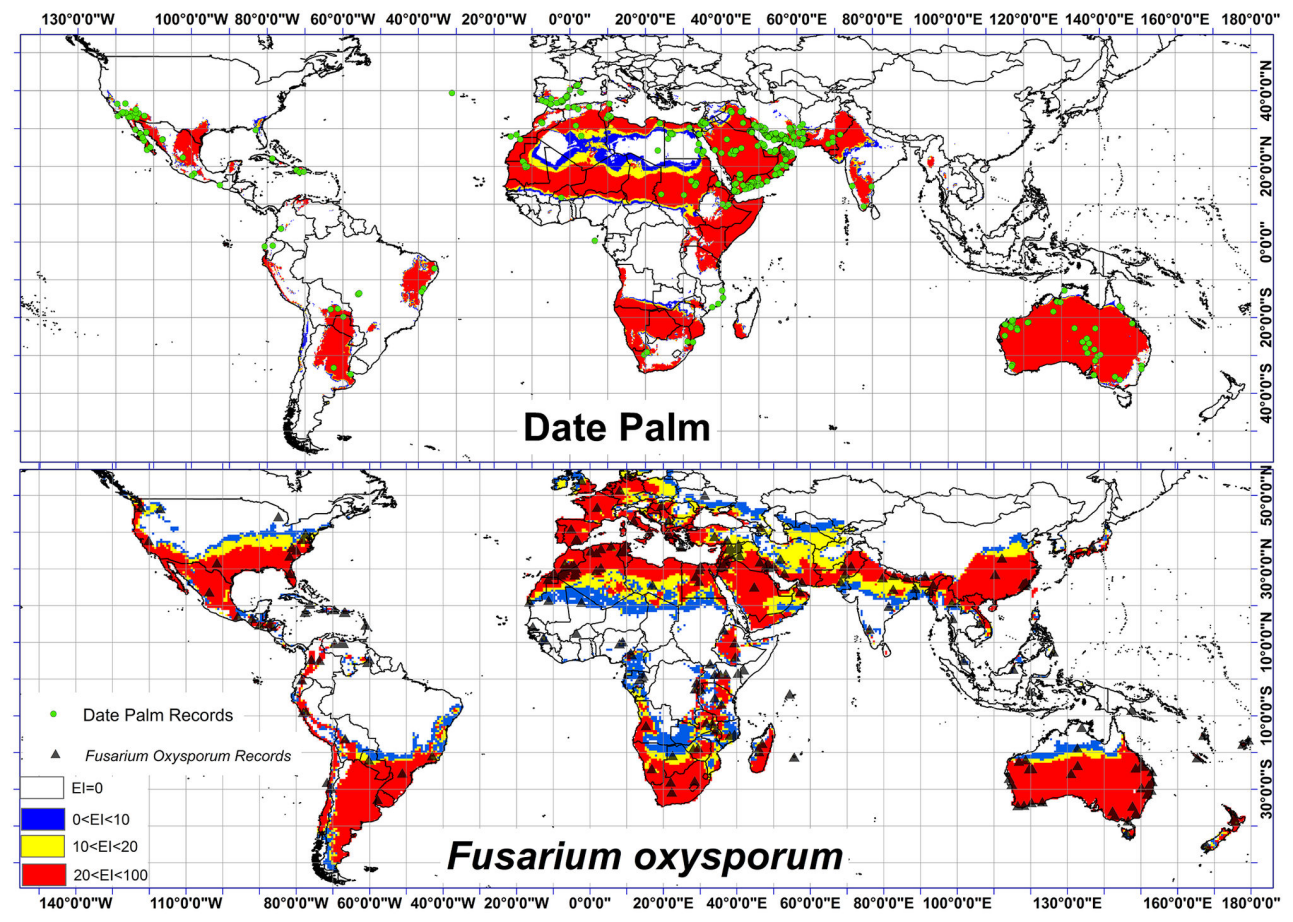
Location of date palm cultivation and *Fusarium oxysporum* f.sp. and model validations for both species under the current climate.

The Global Biodiversity Information Facility (GBIF) [47], Atlas of Living Australia [48], Australia's Virtual Herbarium [49], Discover Life [50] and other *Fusarium oxysporum* f.sp. literature [11,51-56] in CAB Abstracts databases were used to collect data on the current distribution of *Fusarium oxysporum* f.sp.. In total, 734 records for *Fusarium oxysporum* f.sp. were obtained from this literature and the databases. 340 records did not have geographical coordinates and were removed. Besides, 139 records were duplicates and thus also removed. The remaining 255 records were used in parameter fitting. The distribution of *Fusarium oxysporum* f.sp. at a global scale is shown in Figure 1.

### CLIMEX Software

Projecting potential distribution of invasive species is most critical and useful to relevant decision-making at the commencement of an invasion, and in this regard CLIMEX models have proven extremely suitable and robust [57]. This software product assumes that climate is the chief determinant of the distribution of plants and poikilothermal animals and facilitates the modeling of climatic conditions under varied scenarios for various species in ecological studies [58]. The program allows a user to establish climatic parameters that describe the responses of a species to climate, through geographical determinants [59]. Thus the limitation mechanisms imposed on the geographical distribution of a species, its seasonal phenology and, to a lesser extent, its relative abundance may be determined [44]., The Annual Growth Index (GI_A_) incorporated into CLIMEX denotes favorable season potential for population growth throughout the species. The cold, hot, wet and dry stress indices and their interactions illustrate the extent of unfavorable season to population reduction [44]. The Ecoclimatic Index (EI) is derived by multiplying the GI_A_ into the given Stress Indices, establishing the overall measure of the location or year’s favorability for permanent target species’ occupation. This EI, based on weekly calculations of growth and stress indices, thus forms an average yearly index of the level of climatic appropriateness, on a scale from 0 to 100, such that an EI > 0 denotes potential for establishment of the species. CLIMEX was used, in this study, for creating the model for potential distribution of *Fusarium oxysporum* f.sp. under current and selected future climate scenarios. Climatic parameters from 1950 to 2000, included in the CliMond 10’ gridded climate records [44], were averaged in order to model the current *Fusarium oxysporum* f.sp. distribution. Climatic parameters utilized from the meteorological database were overall maximum and minimum monthly temperature (*T*_*max*_ and *T*_*min*_), the overall monthly precipitation (*P*_*total*_) and the relative humidity at 09:00 h (RH_*09:00*_) and 15:00 h (RH_*15:00*_). These parameters also formed the basis for projections of potential climatic conditions for the respective years selected between the mid- and late 21^st^ century.

### Global Climate Models and Climate Change Scenarios

Two Global Climate Models (GCMs), namely, CSIRO-Mk3.0 (CS) and MIROC-H (MR), with the A2 Special Report on Emissions Scenarios (SRES) scenario were selected to model potential distribution of *Fusarium oxysporum* f.sp. under future climates in this study. The A2 SRES scenario was selected in that it incorporates relevant demographic variables and financial and technological factors relating to greenhouse gas (GHG) emissions. A2 scenario assumptions include population and regional economic development data derived from independent, self-reliant nations [60]. A2 scenario predictions show a relatively moderate increase in GHG, approximating the midpoint of extreme low and high projections. The A2 SRES scenario was used for the selected study years of 2030, 2050, 2070 and 2100. 

### Model Framing

Using distribution data derived from different sources on *Fusarium oxysporum* f.sp., a CLIMEX model was built depicting its climate suitability. In this model, stress values were adjusted depending on satisfactory agreement between the potential and known distribution of *Fusarium oxysporum* f.sp. at the global scale. 

#### • Wet Stress

Environmental and soil conditions are important for infection and in symptom expression. Oritsejafor [61] suggests that survival of the *Fusarium oxysporum* f.sp. fungus decreases with increases in soil moisture. The highest survival rate of the pathogen was recorded at the lowest tested level of soil moisture. Oritsejafor further states that *Fusarium oxysporum* f.sp. is highly sensitive to soil moisture level fluctuation. Therefore, the values of wet stress in CLIMEX should be high to cover this sensitivity. The wet stress threshold (SMWS) was set to 9 out of 10 and the accumulation rate (HWS) was set at 0.001 week^–1^ to allow this species to establish in areas in African countries where there are known current records. 

#### • Dry Stress

Moisture stress impacts upon a species when conditions are too dry or too wet. A soil moisture level falling below the Dry Stress Threshold (SMDS) indicates Dry Stress. The SMDS and soil moisture level (SM) difference is then multiplied by the Dry Stress Rate (HDS) and the product denotes the resultant Dry Stress for the week [44]. It is reported by Ghaemi et al. [62] that *Fusarium oxysporum* f.sp. reacts sensitively to moisture, and that disease symptoms appear quicker in treatments with water stress, than with other treatments. Thus values representing dry stress should be low, and consequently the dry stress threshold (SMDS) was set to 0.007 and the dry stress rate was set at -0.009 week^–1^. 

#### • Cold Stress

The cold stress temperature threshold (TTCS) and cold stress temperature rate per week (THCS) are the parameters indicating cold stress in the CLIMEX software. TTCS defines a temperature below which cold stress begins to accumulate and THCS describes the rate at which this stress accumulates. Robinson et al. [63] demonstrate that hyphae of *Fusarium oxysporum* f.sp. can resume their growth at 4°C. This intolerance to frost was incorporated by setting the accumulation stress when the average monthly minimum temperature fell below 4°C, with the frost stress accumulation rate (THCS) set at 10^-3^ week^-1^. The cold stress temperature threshold (TTCS) was set at 10^-4^ to allow this species to establish in areas where *Fusarium oxysporum* f.sp. exists in the United States, China and Turkey.

#### • Heat Stress

In CLIMEX, TTHS and THHS define the heat stress parameter and heat stress accumulation rate respectively. The TTHS parameter was set at 28°C in that it is documented that *Fusarium oxysporum* f.sp. is capable of persisting in conditions up to this temperature [64]. The heat stress accumulation rate (THHS) was set at 10^-6^ week^–1^, which allows for the persistence of *Fusarium oxysporum* f.sp. in southern Iran, Saudi Arabia, Oman, Egypt, Pakistan, and Australia. 

#### • Growth Index

The growth index in CLIMEX software is obtained by multiplying the temperature and moisture indices. The temperature index comprises of DV0: limiting low temperature, DV1 and DV2: lower and upper optimal temperatures respectively, and DV3: the limiting high temperature. *Fusarium oxysporum* f.sp. is found in areas with temperatures between 18° and 28°C [65,66]. Data records with climatic conditions appropriate for *Fusarium oxysporum* f.sp. growth show that actual conditions in Australia, Mozambique, Madagascar, Morocco and Spain match these requirements. Thus, for this species, the limiting low temperature (DV0) should be around 18°C. 16°C was determined to provide the best fit for the observed distribution of *Fusarium oxysporum* f.sp. in Iran, South Africa, Eastern Brazil, Mexico and Colombia. It has been reported that *Fusarium oxysporum* f.sp. activities decline dramatically at 28°C [65]. Thus, the DV3 was set at 28°C. The DV1 was set at 17°C as it has been documented to be a suitable temperature for *Fusarium oxysporum* f.sp. growth [65]. The DV2 was set at 25°C since Saremi et al. [67] conclude that temperatures up to 25°C are suitable for *Fusarium oxysporum* f.sp. development. Maximum values were set for parameters DV1, DV2 and DV3 so that maximum values, within native areas with the highest density of records, were exhibited by the EI. 

The soil moisture index in CLIMEX consists of the lowest threshold (SM0), the lower (SM1) and upper (SM2) optimum moisture thresholds, and the upper threshold (SM3). In this analysis SM0 was set at 10^-5^ to represent the permanent wilting point [68]. This value fits the observed distribution of *Fusarium oxysporum* f.sp. in Senegal, Mauritania, Algeria, Kenya, Tanzania and Namibia. The lower (SM1) and upper (SM2) optimum moisture limits were set at 0.008 and 8, respectively, to include *Fusarium oxysporum* f.sp. distribution in the United States, Cuba, Puerto Rico, Venezuela, Guyana and Turkey. The upper soil moisture threshold (SM3) was set at 9 as it has been established that this pathogen can be negatively affected by high soil moisture [64,69]. The selected values for parameters SM0, SM1, SM2 and SM3 allowed the highest EI values within areas that had the highest density of records. Table 1 provides a summary of all CLIMEX parameters.

**Table 1:** The CLIMEX parameter values that were used for *Fusarium oxysporum* f.sp. modeling.

Index	Parameter	Mnemonic	Values
	Limiting low temperature	DV0	16°C
Temperature	Lower optimal temperature	DV1	17°C
	Upper optimal temperature	DV2	25°C
	Limiting high temperature	DV3	28°C
	Limiting low soil moisture	SM0	10^-5^
Moisture	Lower optimal soil moisture	SM1	0.008
	Upper optimal soil moisture	SM2	8
	Limiting high soil moisture	SM3	9
Cold Stress	Cold stress temperature threshold	TTCS	0.0001
	Cold stress temperature rate	THCS	-0.001 week^–1^
Heat Stress	Heat stress temperature threshold	TTHS	28°C
	Heat stress accumulation rate	THHS	10^-6^ week^–1^
Dry Stress	Dry stress threshold	SMDS	0.007
	Dry stress rate	HDS	-0.009 week^–1^
Wet Stress	Wet stress threshold	SMWS	9
	Wet stress rate	HWS	0.001 week^–1^

### Combination of both GCMs outputs for *Fusarium oxysporum* f.sp. and date palm

Agreement in projections of suitable areas for *Fusarium oxysporum* f.sp. growth from CS and MR GCMs were overlaid to identify common areas that were projected to be highly conducive to *Fusarium oxysporum* f.sp. presence for 2030, 2050, 2070 and 2100. A similar task was undertaken to identify highly conducive areas for date palm as predicted by both the GCM models. These two resulting layers were then overlaid to categorize different risk levels of *Fusarium oxysporum* f.sp. for the predicted future distribution of date palms. All locations that satisfied the condition of EI < 10 for *Fusarium oxysporum* f.sp. and EI > 20 for date palm, from both GCMs, were selected and extracted as areas potentially suitable for date palm cultivation, with low risk of invasive *Fusarium oxysporum* f.sp. growth. The condition with 10 < EI < 20 for *Fusarium oxysporum* f.sp. requirement and EI > 20 for date palm cultivation were identified as areas suitable for date palm cultivation but with a marginal risk of invasive *Fusarium oxysporum* f.sp.. Finally, areas with EI > 20 for both species were extracted and defined as areas highly conducive for date palm but with high risk of *Fusarium oxysporum* f.sp.. 

## Results

### • Historical Climate

Comparing the global climate suitability model with global distribution of *Fusarium oxysporum* f.sp. (Figure 1) shows the consistent correlation of the modeled EI with the current global distribution of *Fusarium oxysporum* f.sp.. Climatic conditions suitable for *Fusarium oxysporum* f.sp. are projected for the eastern, southern and western areas of the United States, Mexico, Guatemala, western Colombia, small areas in central regions of Venezuela, western Peru, southern Brazil, Uruguay, Argentina and Chile. Similar conditions are also projected for Morocco, Egypt, Algeria, Libya, South Africa, Madagascar, Namibia, Botswana, western Ethiopia and most of European countries. Suitable climatic conditions for invasive *Fusarium oxysporum* f.sp. are projected in southern Iran, Saudi Arabia, northern India, eastern China and large parts of Australia. The fact that nearly 87% of the *Fusarium oxysporum* f.sp. records fell within the modeled suitable global climate confirms that the values selected for the various parameters in CLIMEX were optimum (Figure 1). The result of the same modeling for date palm by Shabani et al. [28] indicates that 93% of date palm records fell within the modeled suitable global climate. Refer to Shabani et al. [28] for a detailed discussion of the worldwide projected distribution of date palm based on future climate scenarios.

### • Projected result from the CS model for *Fusarium oxysporum* f.sp.

**Figure 2:**
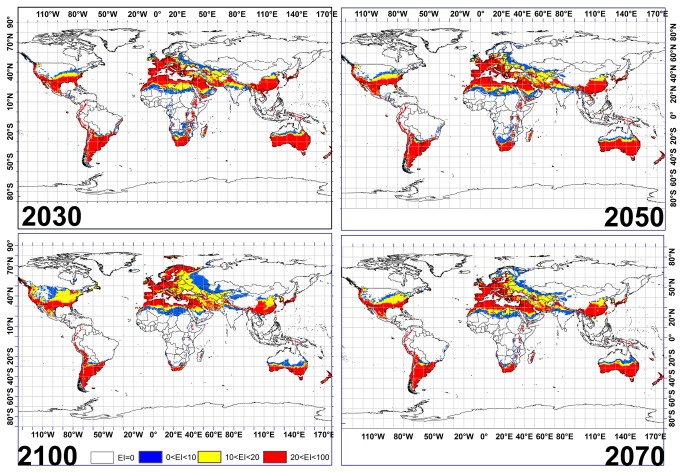
The climate (EI) for *Fusarium oxysporum* f.sp. using CLIMEX under the CSIRO-Mk3.0 GCM running the SRES A2 scenario for 2030, 2050, 2070 and 2100.

According to projected scenarios for 2030, 2050 and 2070, greater portions of Australia are projected by the CS modeled outputs to become particularly suitable for *Fusarium oxysporum* f.sp. growth (Figure 2). However, this model demonstrates that this suitability from 20° to 30° S and 115° E to 160° E will decline significantly between 2070 and 2100 (Figure 2). Similar trends also were projected for eastern China, where unsuitability will begin from 2070. Conversely, this model indicates that large areas in eastern and southern African countries such as Kenya, Tanzania, Mozambique, Zimbabwe, Botswana and Namibia will become significantly unsuitable for the development of this invasive species from 2030 to 2100. Noticeably, many European countries such as Spain, Germany, France, Italy Hungary and the Netherlands, as well as some northern Africa countries, are projected to remain suitable for *Fusarium oxysporum* f.sp. occupation up to 2100. Interestingly, the CS model projects that Sweden and western Finland will become suitable for this invasive species from 2100. It should be highlighted that this model shows that most regions located between 20° S to 20° N will become unsuitable for *Fusarium oxysporum* f.sp. from 2030, with the exception of Ecuador, Chile, and eastern Peru (Figure 2). 

### • Projected result from the MR model for *Fusarium oxysporum* f.sp.

**Figure 3:**
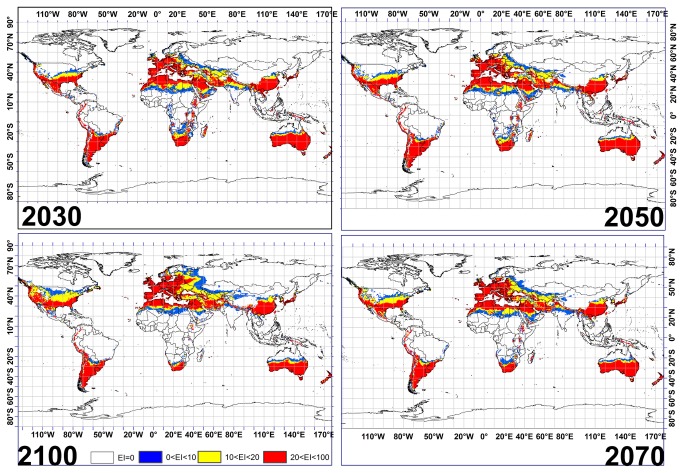
The climate (EI) for *Fusarium oxysporum* f.sp. using CLIMEX under the MIROC-H GCM running the SRES A2 scenario for 2030, 2050, 2070 and 2100.

Figure 3 illustrates the Ecoclimatic Index for *Fusarium oxysporum* f.sp., using the MR global climate change model with the A2 emission scenarios for 2030, 2050, 2070 and 2100. Based on the output of the MR GCM, large areas located from 110° W to 120° W and 30° N to 40° N in the United States are projected to become highly suitable for *Fusarium oxysporum* f.sp. growth between 2030 and 2100. This model indicates that Sweden and Finland may never become suitable for this invasive species growth before 2100, since unsuitable climatic conditions may prevail. It should be pointed out that this model projects that areas in western Russia may become highly suitable for *Fusarium oxysporum* f.sp. by 2100. The MR results also show an upward trend in the unsuitability of areas for *Fusarium oxysporum* f.sp. growth in most of the countries located in central and eastern Africa. However, the indication for all countries located in northern Africa including Morocco, Algeria, Tunisia, Northern Libya and northern Egypt is that the climatic condition may remain suitable for growth of this invasive species. 

###  Summary of CS and MR results for date palm reported by Shabani et al. [28]

**Figure 4:**
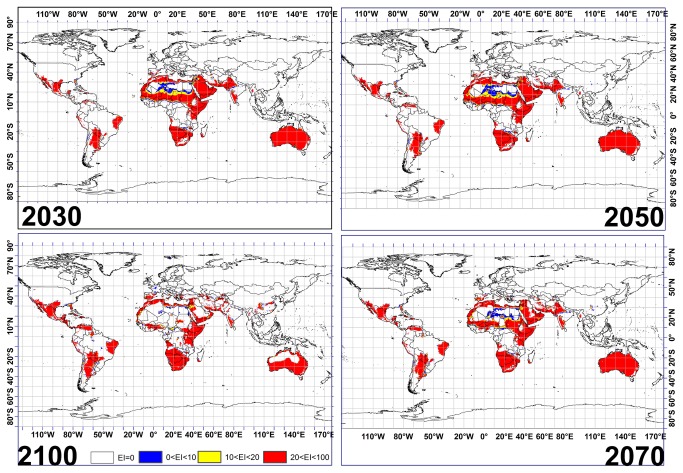
The climate (EI) for *P. dactylifera* using CLIMEX under the CSIRO-Mk3.0 GCM running the SRES A2 scenario for 2030, 2050, 2070 and 2100. This result is taken from Shabani et al [28].

**Figure 5:**
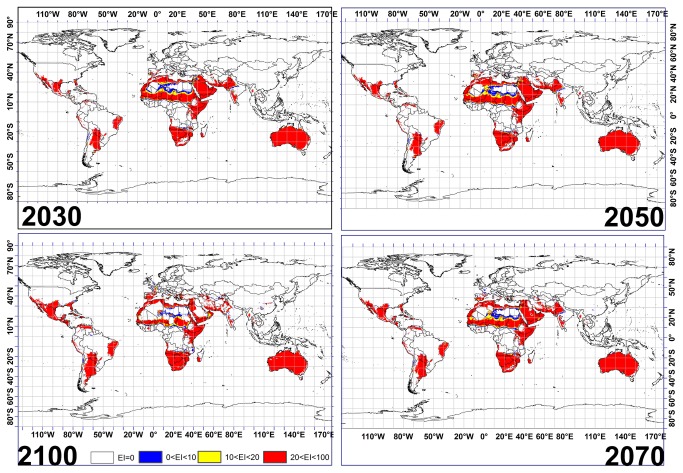
The climate (EI) for *P. dactylifera* using CLIMEX under the MIROC-H GCM running the SRES A2 scenario for 2030, 2050, 2070 and 2100. This result is taken from Shabani et al [28].

On the American continents, in much of the south-western coast of Mexico and North America, eastern Brazil, south-eastern Bolivia, northern Venezuela, Cuba, northern Colombia, and Paraguay, the CS GCM model projects greater climatic suitability for *P. dactylifera* by 2030, increasing consistently through 2050 and 2070 to 2100 (Figure 4). In the Middle East the CS GCM shows that Saudi Arabia, Iraq and western Iran should become climatically suitable by 2030, (Figure 4). By 2050 however in all of these three countries, reduced suitability of the climate for *P. dactylifera* will occur, a trend most accentuated in Saudi Arabia and Iraq around 2100 (Figure 4). The MR GCM output confirms that the same areas on the North and South American continents are projected to become climatically suitable for growth of date palm between 2030 and 2100 (Figure 5). However, the MR GCM also shows areas around Florida that will become suitable for growth of the species by 2100 (Figure 5). Additionally, the MR GCM also shows that western Argentina will be more climatically suitable than currently for *P. dactylifera* growth. Some differences in the projection of date palm distribution are shown in a comparison of the CSIRO-Mk3.0 and MIROC-H GCM results. Differences in predicting future climate is the reason for the differing results in the two GCMs. 

### • Combining the results of both GCM outputs for *Fusarium oxysporum* f.sp. and date palm

**Figure 6:**
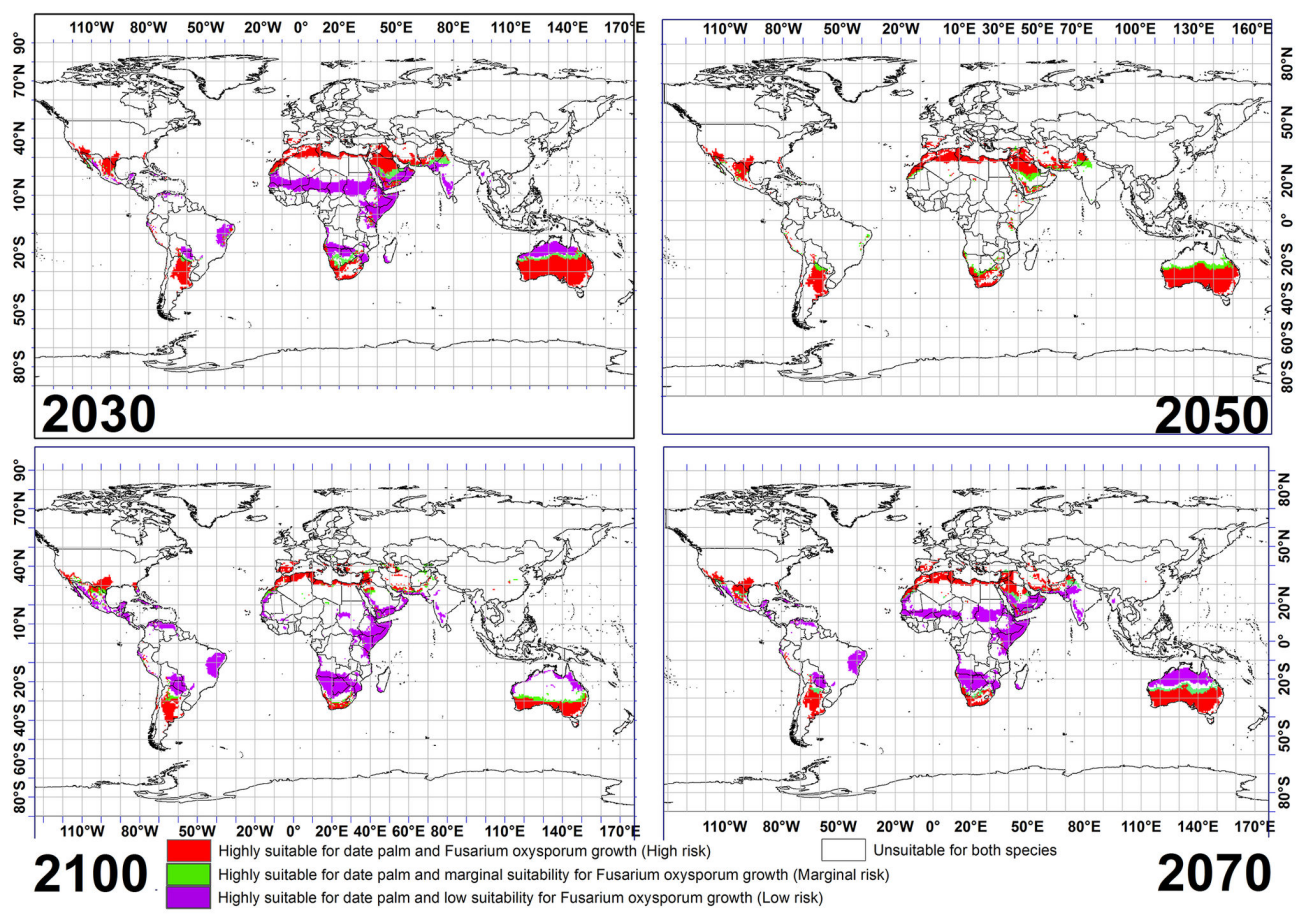
Agreement in the CLIMEX projection of suitable areas for *P. dactylifera* growth with three risk levels of invasive *Fusarium oxysporum* f.sp. under CSIRO-Mk3.0 and MIROC-H GCMs running the SRES A2 scenario for 2030, 2050, 2070 and 2100 based on EI for both species.

Combining the CS and MR GCMs results for both species shows that northern Australia will be highly suitable for date palm cultivation with low risk of *Fusarium oxysporum* f.sp. until 2070 (Figure 6). Similar conditions are also projected for Sudan, Niger, Saudi Arabia, Mali and Mauritania. This result shows that most regions between 10° N and 20° N in Africa will be highly conducive to date palm with low risk from *Fusarium oxysporum* f.sp until 2070 (Figure 6). However, this suitability for regions between 10° N and 20° N in Africa and northern Australia is projected to shrink drastically by 2100. Further, areas in southern Angola, Namibia, Botswana, eastern Brazil, eastern Bolivia and northern Venezuela are projected to have low risks from *Fusarium oxysporum* f.sp. for date palm cultivation from 2030 to 2100 (Figure 6). 

When overlaying the CS and MR GCM results for date palm and *Fusarium oxysporum* f.sp., projections indicate that southern Australia, southern Iran, most of the northern regions in all North African countries, Spain, southern regions of the United States, northern Argentina, and South Africa will become at high risk from *Fusarium oxysporum* f.sp. for cultivation of date palm from 2030 to 2100 (Figure 6). Moreover, this model demonstrates that western Syria and Israel will become suitable for date palm cultivation, albeit at a high risk level of *Fusarium oxysporum* f.sp. by 2100 (Figure 6). 

Overlaying the CS and MR GCM results for date palm and *Fusarium oxysporum* f.sp. indicates that a number of the countries projected to be highly conducive to date palm will be under high risk of *Fusarium oxysporum* f.sp. while many others will be at low risk (Figure 6). However, our result also shows that the southern region of Saudi Arabia, northern India and regions between 25° S and 20° S in Australia will be under marginal risk for date palm cultivation by 2030 and 2050 (Figure 6). Also, results of this study indicate a southern shift in areas conducive to date palm under marginal risk of *Fusarium oxysporum* f.sp., in Australia from 2070 to 2100.

## Discussion

Although CLIMEX modeling outputs do not consider different GCMs and scenarios simultaneously, other software such as ArcGIS can play an important role in finding these areas of agreements and disagreements. This study made use of the ArcGIS software to find the agreements between the results of different GCMs and to extract the location of areas becoming highly conducive to date palm under different risk levels of invasive *Fusarium oxysporum* f.sp. under different GCMs, from 2030 to 2100. 

The CS and MR GCMs share some similarities in the projections of suitable areas for *Fusarium oxysporum* f.sp. or date palm growth. However, inconsistencies between the results of the CS and MR GCMs were also found (Figures 2 to 5). For example, the CS model projects that most regions located between 25 °S and 20 °S in southern Africa will have 0 < EI < 10 for *Fusarium oxysporum* f.sp., while the MR model indicates EI between 10 and 20 for 2030. Similar disagreements can be found in regions located between 20 °S and 25 °S in Australia for 2030 onwards. Additionally, a comparison of the CS and MR GCM results revealed minor disagreements in Latvia, Estonia, Belarus and Lithuania for *Fusarium oxysporum* f.sp. Similar inconsistencies for date palm can be seen in regions located between 0° and 40° E and 20° N and 35° N between 2050 and 2100. Interestingly, the disagreements between the CS and MR GCM outputs for highly conducive areas for date palm cultivation showed a difference of 29% in area by 2100 (Figures 4 and 5). These results show that the difference in projected areas conducive to both species could be considerable (Figures 2 to 5), and can be attributed to the differences in the two models, in rainfall and temperature projected rates of change, as the temperature and rainfall changes predicted by the two GCMs differ. In the case of the MR model a temperature increase of 4.31°C is predicted, as opposed to 2.11°C in the CS model, by 2100 [70,71]. Regarding rainfall, predictions differ between the two models, with the CS predicting a 13 to 15% decrease, whereas the MR predicts only a 1% reduction in future annual rainfall [70,71]. 

We considered the future distribution of the *Fusarium oxysporum* f.sp. threat and one of its most important hosts, date palm, in different GCMs. Since there are large uncertainties in the projections generated by GCMs [72, 73], by finding the agreements in results of different GCMs we have reduced the uncertainties in assessing the impact of climate change on future climatic projections derived by GCMs.

**Figure 7:**
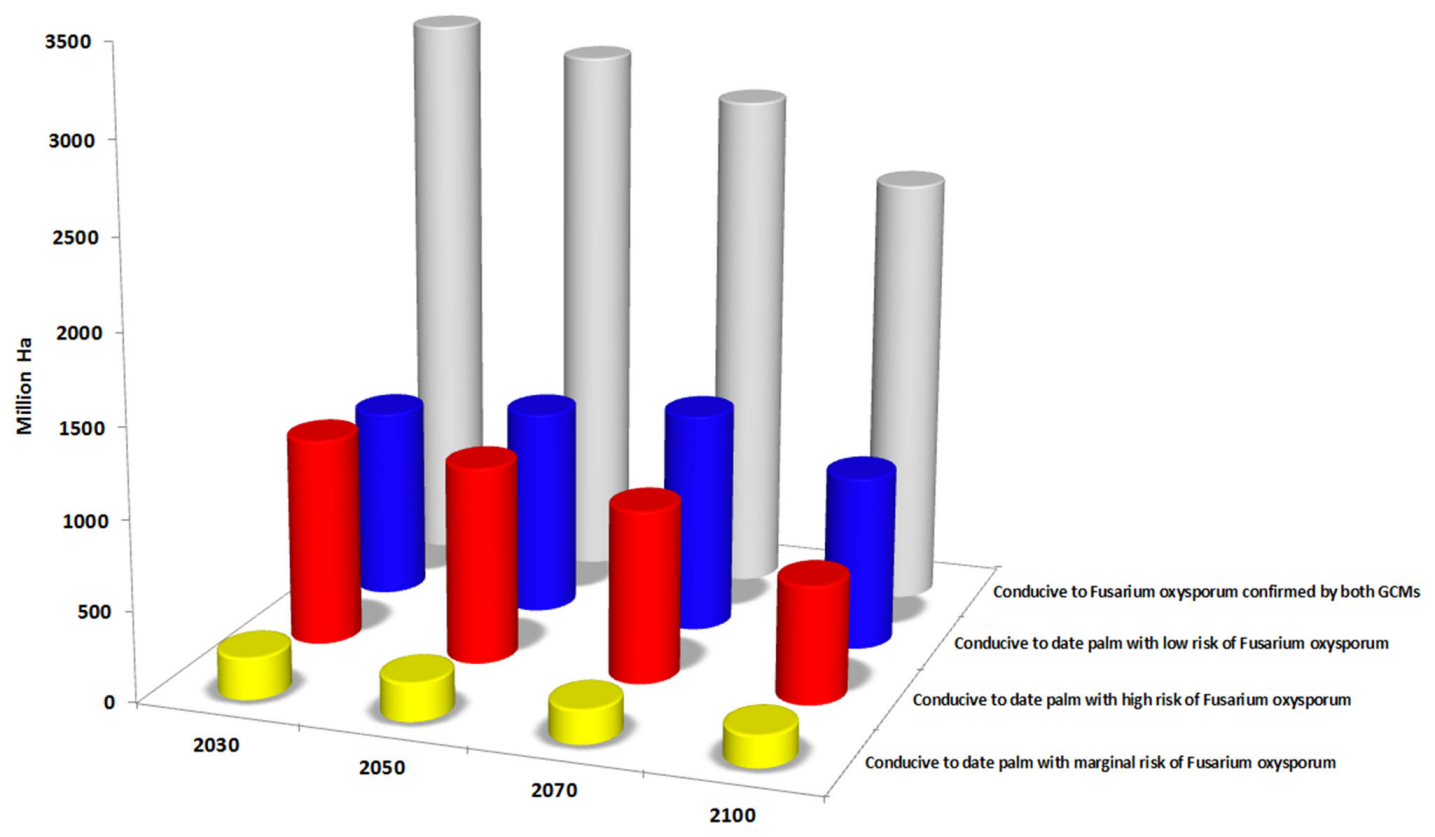
The combination result of CS and MR GCMs for *Fusarium oxysporum* f.sp. and *P. dactylifera* for 2030, 2050, 2070 and 2100 in million hectares based on the Ecoclimatic Index.

The results of GCMs for a single species have the potential to be refined using the distribution of other species that have symbiosis or a parasite relationship with it. This can provide informative data to governments and people in related industries, in developing long-term management strategies to sustain production, especially in the case of economic crops. For example, our study indicates that the CS and MR GCMs agree that 2852, 2921, 2853 and 2310 million hectares across the globe will become highly conducive to date palm cultivation by 2030, 2050, 2070 and 2100 respectively while considering climatic factors alone. However, 40%, 37%, 33% and 28% of these areas will be at high risk of invasive *Fusarium oxysporum* f.sp. for 2030, 2050 2070 and 2100 (Figure 7). As already mentioned, this fungus was able to kill two-thirds of all palm trees (more than 10 million trees) in Morocco and Algeria in 20^th^ centuries. Thus projections forecast here can be used to make informed decisions about future expansion and risk of date plantations. 

We find a projected downward trend for areas that will become highly conducive to date palm growth with high risk of *Fusarium oxysporum* f.sp. (Figure 7), however, we see an upward projected trend for areas with low risk of the fungus until 2070 and then there is a slight decline to 2100 (Figure 7). The reason for this decline from 2070 to 2100 is that the overall area conducive for date palm declines significantly as well. Therefore, governments of countries projected to become highly conducive to date palm with marginal or low risk of *Fusarium oxysporum* f.sp., should make use of this advance information, to develop and implement appropriate policies and programs that help to assure the date palm industry’s safety, and developing a date palm biosecurity system. Strong policies in transferring soil, seed and water from one country or one continent to another can help governments to take the initial steps necessary to protect their agricultural products, since there exists a body of literature showing that the disease can be easily spread by soil attached to machinery, agricultural tools, water, and plant debris from field to field, or within a region or country. 

## Conclusion

Accurate projections of land use evaluation and climatic modeling are vital for effective long-term planning of agricultural production. Date palm production agronomists and planners may effectively utilize the modeling and refinement of our results to take timely action to minimize adverse impacts indicated by the projected future scenarios. Distribution maps similar to those illustrated in this study may also be of value in formulating more cost-effective future production methods for agricultural crops. Maps such as that shown in Figure 6 can provide vital information on change in localities suitable for date palm cultivation under different risk levels of *Fusarium oxysporum* f.sp., enabling plantation owners and managers to consider the long-term implications of current management decisions. The methods utilised in this study also have relevance and application for other agricultural crops. The results of the climate change for two different species within two different GCMs provides an indication of the probable changes in the location of cash crop farming and associated industries. The results indicate that more areas with low risk of invasive *Fusarium oxysporum* f.sp. will be available in areas projected to become highly conducive to date palm cultivation until 2070. However, a downward trend was projected in areas highly conducive to date palm and *Fusarium oxysporum* f.sp. by 2030, 2050, 2070 and 2100. Further research could consider non-climatic factors in addition to CLIMEX-based modeling, by overlaying further layers on a map derived from CLIMEX, to refine the results in a range of environments and locations. 
